# The Association of HIV-1 Neutralization in Aviremic Children and Adults with Time to ART Initiation and CD4+/CD8+ Ratios

**DOI:** 10.3390/vaccines12010008

**Published:** 2023-12-20

**Authors:** Victor Sanchez-Merino, Miguel Martin-Serrano, Manuela Beltran, Beatriz Lazaro-Martin, Eloisa Cervantes, Manuel Oltra, Talia Sainz, Felipe Garcia, Maria Luisa Navarro, Eloisa Yuste

**Affiliations:** 1 National Microbiology Center, Institute of Health Carlos III (ISCIII), 28220 Madrid, Spain; mmartin.imas12@h12o.es (M.M.-S.); mbeltran@isciii.es (M.B.); 2 Faculty of Health Sciences, Alfonso X el Sabio University, 28691 Madrid, Spain; 3 Centro de Investigación Biomédica en Red de Enfermedades Infecciosas (CIBERINFEC), 28029 Madrid, Spain; talia.sainz@salud.madrid.org (T.S.); marisa.navarro.gomez@gmail.com (M.L.N.); 4 Department of Medical Oncology, Hospital Universitario 12 de Octubre, Instituto de Investigación Sanitaria Hospital 12 de Octubre (imas12), 28041 Madrid, Spain; 5 Servicio de Pediatría, Hospital General Universitario Gregorio Marañón, 28009 Madrid, Spain; bealamartin@gmail.com; 6 Sección de Infectología Pediátrica, Hospital Clínico Universitario Virgen de la Arrixaca, 30120 Murcia, Spain; eloisacervantes@gmail.com; 7 Sección de Patologia Infecciosa Infantil, Hospital Universitari i Politècnic La Fe, 46026 Valencia, Spain; oltra.benavent@gmail.com; 8 Department of Pediatrics, Infectious and Tropical Diseases, La Paz Research Institute (IdiPAZ), La Paz University Hospital, 28046 Madrid, Spain; 9 Facultad de Medicina, Universidad Autónoma de Madrid, 28049 Madrid, Spain; 10 Infectious Diseases Department, Hospital Clínic, University of Barcelona, 08036 Barcelona, Spain; fgarcia@clinic.cat

**Keywords:** HIV-1, broadly neutralizing antibodies, children, ART, undetectable viremia

## Abstract

Broadly neutralizing antibodies (bnAbs) bind and neutralize diverse HIV isolates and demonstrate protective effects in primate models and humans against specific isolates. To develop an effective HIV vaccine, it is widely believed that inducing these antibodies is crucial. However, the high somatic hypermutation in bnAbs and the limited affinity of HIV Env proteins for bnAb germline precursors suggest that extended antigen exposure is necessary for their production. Consequently, HIV vaccine research is exploring complex sequential vaccination strategies to guide the immune response through maturation stages. In this context, the exploration of the factors linked to the generation of these antibodies across diverse age groups becomes critical. In this study, we assessed the anti-HIV-1 neutralization potency and breadth in 108 aviremic adults and 109 aviremic children under 15 years of age who were receiving ART. We used a previously described minipanel of recombinant viruses and investigated the factors associated with neutralization in these individuals. We identified individuals in both groups who were capable of neutralizing viruses from three different subtypes, with greater cross-neutralization observed in the adult group (49.0% vs. 9.2%). In both groups, we observed an inverse association between neutralization breadth and the CD4+/CD8+ ratio, as well as a direct association with the time to ART initiation. However, we found no association with time post-infection, cumulative ART duration, or CD8+ cell levels. The present study demonstrates that children receiving antiretroviral therapy generate broadly neutralizing responses to HIV-1, albeit with lower magnitude compared to adults. We also observed that neutralization breadth is associated with CD4+/CD8+ levels and time to treatment initiation in both children and adults living with HIV-1. Our interpretation of these results is that a delay in ART initiation could have prolonged the antigenic stimulation associated with viral replication and thus facilitate the capacity to elicit long-lasting broadly neutralizing responses. These results corroborate prior findings that show that HIV-1-neutralizing responses can persist for years, even at low antigen levels, implying an HIV-1 vaccine may induce lasting neutralizing antibody response.

## 1. Introduction

Despite the significant advances made with combination antiretroviral therapy (ART), which have resulted in reduced mortality and increased lifespan for individuals living with HIV, it does not offer a cure [1]. Patients are obligated to remain on ART for life, suffering from accompanying side effects, the financial burden of long-term treatment, and the development of drug resistance [2]. Consequently, there is a compelling need for an HIV vaccine.

In the case of approved antiviral vaccines, neutralizing antibodies play a crucial role [3]. However, despite extensive efforts, attempts to design an HIV-1 vaccine capable of eliciting protective antibody responses have been unsuccessful thus far. In natural HIV-1 infections, evolving virus strains challenge neutralizing antibodies, limiting their effectiveness to the individual’s specific virus strain, with minimal cross-strain neutralization [4,5]. The discovery of bnAbs has brought renewed hope to HIV-1 vaccine development over the last decade, as they possess the remarkable ability to neutralize a wide range of HIV-1 strains, spanning various subtypes. Indeed, certain bnAbs have shown promising protective effects in both primate models and humans when exposed to specific viral isolates [6,7]. As a result, the prevailing belief in the scientific community is that the induction of bnAbs is a critical milestone in the quest to develop an effective HIV vaccine. This has required intensive research efforts with the aim of understanding how bnAbs develop in natural HIV-1 infection [8].

In the absence of treatment, the development of neutralization breadth in adults has been associated with several clinical factors, including time since infection, high virus load, the presence of normalized B-cell subpopulations, and early decline in circulating CD4+ T cells [9,10,11]. In addition, the fact that many of the bnAbs exhibit high levels of somatic hypermutation and the low affinity of HIV Env proteins for germline bnAb precursors seems to indicate that the induction of these antibodies requires high maturation times in the presence of the antigen. Furthermore, the substantial somatic hypermutation in many bnAbs and the low affinity between HIV Env proteins and germline bnAb precursors suggest that generating these antibodies demands prolonged maturation in the presence of the antigen, which makes it difficult to design vaccination strategies for the induction of these antibodies [12,13,14].

In the case of HIV-infected children, neutralization breadth develops earlier and at a higher frequency than in adults [15]. Detailed epitope mapping of bnAbs showed that 63% of the children developed polyclonal responses targeting multiple sites on the HIV envelope [16]. This finding contrasts with the prevailing observation in adults, where neutralization breadth is typically driven by antibodies with more limited specificities [17]. This difference could indicate that the mechanisms responsible for achieving neutralization breadth in adults differ from those in children.

One of the factors that may have contributed to the differences observed between the ability to induce bnAbs in children and adults is that, in the early stages of infection, children have much higher viral loads and CD4+ cell counts than adults [18]. In this scenario, antigenic stimulation would be much higher, favoring the activation of multiple B-cell lineages and, therefore, the induction of a broad polyclonal response [16]. On the other hand, during the early stages of life, the immune system exhibits distinctive characteristics that can favor the induction of more robust responses, favoring the amplitude of neutralization. In fact, the high frequency of IL-21-secreting HIV-specific circulating effector T-follicular helper cells in HIV-infected children has been correlated with neutralization breadth [15].

In the case of individuals with ART-associated viral suppression, broadly neutralizing responses have been identified, but these responses decrease dramatically when viral load drops due to ART initiation [11]. However, our understanding of the capacity to produce bnAbs in individuals infected with extended periods of ART and viral load suppression remains limited. A recent study by Schommers et al. showed that a longer time off ART was associated with potent and broad neutralization [19]. Moreover, it is also unknown whether the differences in the ability to induce broadly neutralizing responses observed between adults and children are maintained in the presence of antiretroviral therapy.

In a classical vaccine approach, the induction of long-term broadly neutralizing responses post-immunization without constant booster doses is crucial. The present study examines the factors influencing broadly neutralizing responses in individuals from different age groups with ART-suppressed viremia after initial infection, offering insights for generating bnAbs after long periods of minimal exposure to viral antigens after initial vaccination.

Our findings suggest that prolonged antigenic stimulation before starting ART enhances the ability to generate durable broadly neutralizing responses. Therefore, the induction of these responses without continuous vaccine exposure is feasible, provided the initial antigenic stimulation is substantial.

## 2. Materials and Methods

### 2.1. Study Participants

**Figure 1:**
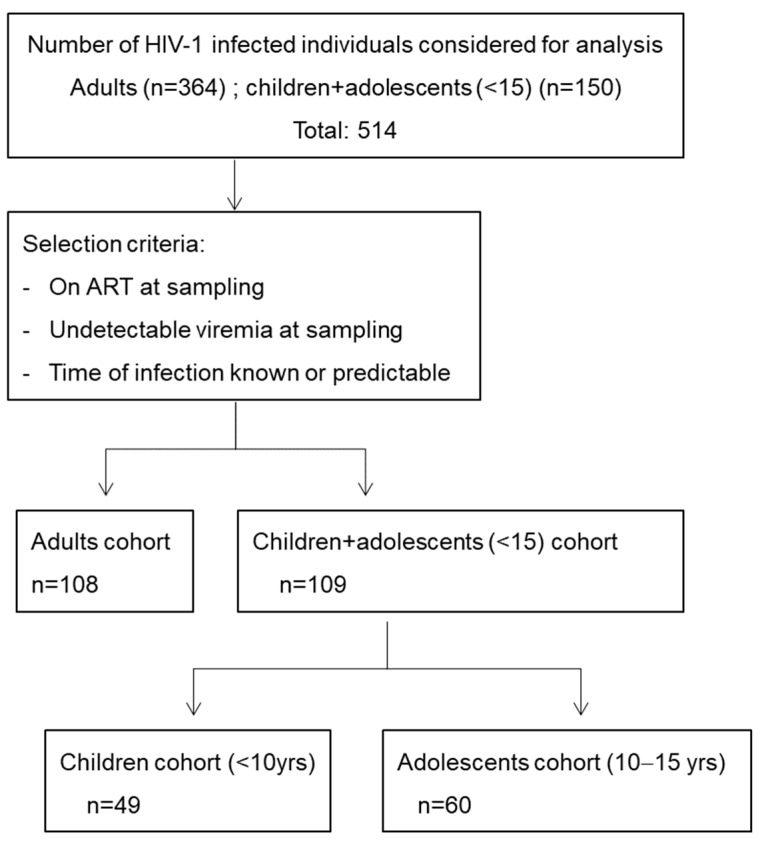
Individual selection and stratification. Plasma or serum-purified IgGs from 514 individuals were included.

For the children and adolescent cohort, 150 plasma samples from individuals aged between 18 months and 15 years were provided by the Spanish Cohort of HIV-Infected Children and Adolescents (CoRISpe [20]). Clinical and immunovirological characteristics were obtained from the CoRISpe’s database. Out of these, 109 were vertically infected and had suppressed viremia due to antiretroviral therapy (ART) at the time of sampling. Adult neutralization data used for comparison were from a previous study [21] including samples from 364 HIV-1-infected individuals treated at the Hospital Clinic of Barcelona, Spain. Among them, 108 had suppressed viremia due to ART at the time of sampling and had known or predictable times of infection (Figure 1).

### 2.2. Neutralization Assays

IgGs from heat-inactivated plasma were purified with protein A columns (GE Healthcare, Chicago, IL, USA) and tested in triplicate at 0.2 µg/mL with a previously described virus minipanel that has previously been used for the identification of cross-neutralizing responses in HIV-1-infected individuals [21,22]. This minipanel consists of six recombinant replication-competent viruses representing five different genetic subtypes: VI 191 (subtype A, tier 2), 92BR025 (subtype C, tier 1B), 92UG024 (subtype D, tier 2), CM244 (subtype AE, tier 2), NL4-3 (subtype B, tier 1A), and AC10.029 (subtype B, tier 2) [11,21,22]. An amphotropic vesicular stomatitis virus (VSV) Env pseudotyped on an HIV-1 core was added to the panel as a specificity control virus in neutralization assays. The neutralization data of the adult cohort used as a comparison were derived from a previous study in which 364 samples were analyzed as described above [21] and, for comparison in the present study, 108 individuals from that cohort with undetectable plasma viremia (<50 viral RNA copies/mL) and known time of infection were selected.

Selected IgGs were tested with an additional pseudotyped virus panel with 10 HIV-1 envs from deCamp panel [23] provided by the NIH HIV Reagent Program (TRO11 (subtype B, tier 2), 25710 (subtype C, tier 2), X2278 (subtype B, tier 2), X1632 (subtype G, tier 2), CE1176 (subtype C, tier 2), 246_F3_C10_2 (CRF A1C, tier 2), 398_F1_F6_20 (CRF A1C, tier 2), BJOX002000_03_2 (CRF BC, tier 2), CH119_10 (CRF BC, tier 2), and CNE55 (CRF AE, tier 2)). Pseudotyped viruses were generated through the cotransfection of an HIV-1 *env* expression plasmid and an *env*-deficient HIV-1 backbone (pNL43ΔenvFL), as previously described [21]. Neutralization assays with pseudotyped viruses were carried out in triplicate at 0.4 µg/mL, and when neutralization percentages greater than 50% were observed, a complete neutralization curve (0.4 to 0.01 µg/mL) was plotted, and the corresponding IC50 values were determined. Thus, we were able to verify that the neutralization values obtained at 0.4 µg/mL in triplicate were similar to those extrapolated from the neutralization curve.

### 2.3. CD4+ and CD8+ T Cell Counts

CD8+ and CD4+ T cells were counted via flow cytometry in whole blood in all cases. However, as a result of the study’s multicenter and retrospective nature, the specific methods used to quantify CD4 and CD8-T cell levels varied across different Spanish hospitals and timeframes. All methods used for the determination of CD4+ and CD8+ T-cell counts were duly certified.

### 2.4. Statistical Analysis

The results are presented as median and range or mean and standard error means (SEM). Mann–Whitney U tests and Kruskal–Wallis test were used for comparisons of two and three groups, respectively. Simple comparisons were made with a two-sided alpha level of 0.05. The association between two variables was determined by Spearman correlation analysis and r and *p* values (two-tailed), and *p*-values < 0.05 were considered statistically significant. Statistical analyses were performed using GraphPad Prism (version 9; GraphPad Software, La Jolla, CA, USA).

## 3. Results

### 3.1. Screening for Cross-Neutralizing Sera in Children and Adolescents

A total of 109 children and adolescents on ART and virologically suppressed were included to screen for cross-neutralizing activity (Figure 1).

**Figure 2:**
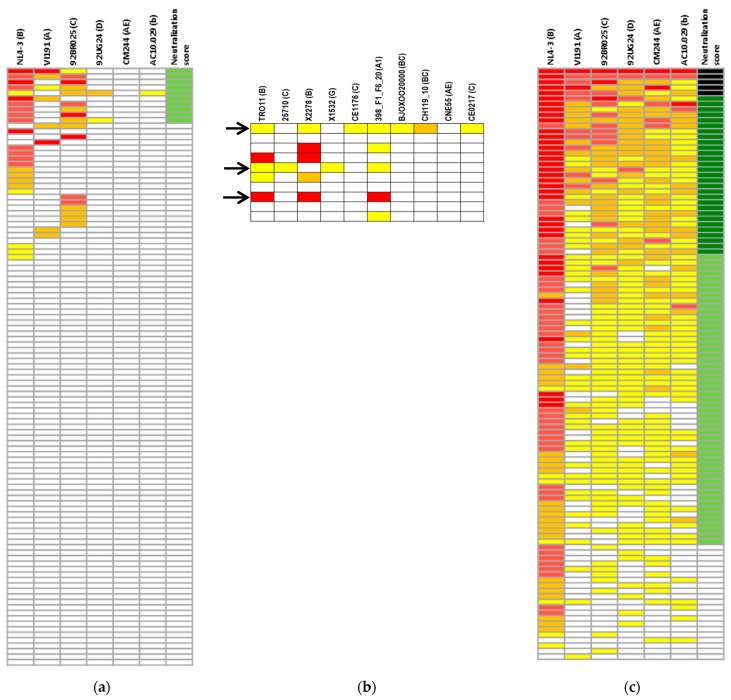
Neutralization data against two virus panels (**a**,**c**). The percentages of neutralization at 0.2 µg/mL plasma-purified IgG and neutralization categories are shown for HIV-1-infected adults (**c**) and children and adolescents (15 years of age) (**a**) against a previously described 6-recombinant virus minipanel [20]. Percentages of neutralization are shown as follows: a white box indicates <20% neutralization; a yellow box indicates ≥20% and <50% neutralization; an orange box indicates ≥50% and <70% neutralization; a light red box indicates ≥70% and <90% neutralization; and a red box indicates >90% neutralization. Neutralization categories are as follows: a black box indicates elite neutralization, a dark green box indicates broad neutralization, a light green box indicates cross-neutralization, and a white box indicates week or no neutralization; (**b**) neutralization data from cross-neutralizer children and adolescent samples identified in the initial screening (identified in light green in (**a**)) against 10 pseudoviruses from the deCamp virus panel [23]. A white box indicates IC50 > 0.4 mg/mL, a yellow box indicates 0.4 ≤ IC50 > 0.2 mg/mL, an orange box indicates 0.2 ≤ IC50 > 0.1 mg/mL, and a red box indicates IC50 ≤ 0.1 mg/mL. Samples that neutralized more than 3 viruses from this panel are indicated with a black arrow.

All of the samples were tested against a previously described six-recombinant virus minipanel. This minipanel includes six viruses from five different subtypes and has previously been used for the identification of cross-neutralizing responses in HIV-1-infected individuals [11,21,22]. IgG neutralization curves were generated for each virus-IgG combination, and the corresponding IC50 values were determined. Neutralization percentages of each purified IgG at a concentration of 0.2 μg/mL against each of the viruses are shown in Figure 2a. To assess neutralization breadth and potency, we used a previously described scoring system based on the percentage of inhibition of each virus of the panel using a single plasma IgG concentration (0.2 µg/mL) [24,25]. In this cohort, the median neutralization score was 1.0 ± 1.8, ranging from 0 to 8. Detectable cross-neutralizing responses, defined as scores of 5–9, were observed in samples from 10 individuals (9.2%). However, we did not identify any individuals with elite (scores of 14–18) or broad (scores of 10–13) neutralizing responses in the children and adolescent cohort (Table 1). No significant neutralization of the VSV-pseudotyped control was observed in any of the purified IgGs analyzed.

Due to the potential interest in broadly neutralizing responses induced in individuals under 15 years of age on ART and with undetectable viremia, we further characterized the neutralization potency and breadth in individuals who showed cross-neutralizing activity in the initial screening using 10 additional pseudotyped viruses from six subtypes and recombinant forms included in the deCamp virus panel [23]. Neutralization results are summarized in Figure 2b. This new panel showed that 3 out of the 10 cross-neutralizers identified in the initial screening neutralized three or more viruses (30.0%) from the new panel (shown in Figure 2b). In this way, we were able to confirm the presence of cross-neutralization in several samples that demonstrated neutralization breadth in the initial screening using a second virus panel.

### 3.2. Neutralizing Antibody Response in Children vs. Adults

Next, we compared the potency and breadth of the IgG neutralizing response in aviremic infected individuals under 15 years of age on ART with the response observed in aviremic adults on ART, similarly determined previously and described in Materials and Methods (Figure 2c) [21]. This comparison has been possible due to the high consistency and reproducibility of the TZM-bl neutralization assay used in both studies, which has been verified previously [26].

**Figure 3:**
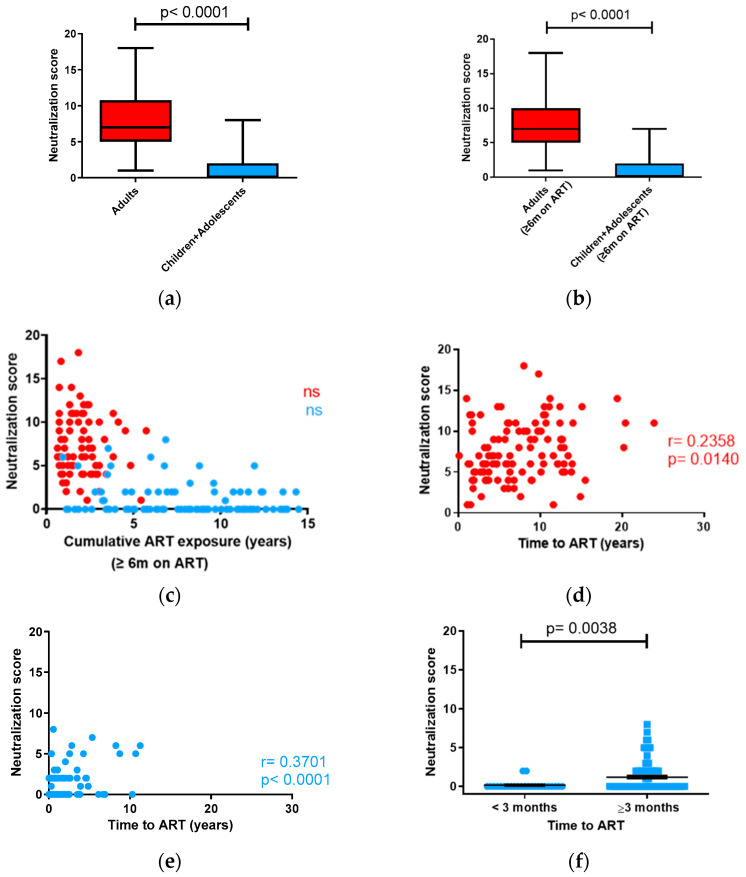
Comparison of neutralizing antibody response (neutralization scores) in adults and individuals < 15 years of age: (**a**) HIV-infected adults and children + adolescents; (**b**) HIV-1-infected adults and children + adolescents on ART for more than 6 months. Horizontal bars within the box plots indicate the median for each group and standard errors of the means (SEMs). Significances between groups are indicated. Mann–Whitney U tests were used for comparisons between groups. Simple comparisons were made with a two-sided alpha level of 0.05; (**c**) correlation of neutralization scores with cumulative ART exposure after the first 6 months of treatment; (**d,e**) correlation of neutralization score values with time to ART initiation in adults (**d**) and children + adolescents (**e**). Spearman r and *p* values (two-tailed) are indicated; ‘ns’ indicates not significant. Adults’ data are indicated in red and children’s data are indicated in blue; (**f**) impact on HIV-1 neutralization in children and adolescents due to delayed ART initiation. Horizontal bars within the point plots indicate the median for each group and standard errors of the means (SEMs). Significance between groups is indicated. Mann–Whitney U tests were used for comparisons between groups. Simple comparisons were made using a two-sided alpha level of 0.05.

Unlike what was observed in the children + adolescent cohort, we were able to identify samples with broad and even elite neutralizing responses in adults. In this cohort, the median neutralization score was 7.6 ± 3.6 ranging from 0 to 14 (Table 1). Adults’ neutralizing profile was as follows: elite neutralizers 4.6%, broad neutralizers 27.0%, and cross-neutralizers 49.0% (Table 1). When comparing both cohorts, we observed significant differences in neutralization breadth, (*p* < 0.0001) (Figure 3a). Next, we analyzed the factors that could be associated with the induction of such responses.

It was previously found that the drop in viral load caused by ART initiation was associated with a decrease in neutralization breadth, but this effect was only observed after 6 months of treatment in all the cases studied [11]. In order to ensure that the differences between both groups observed were not affected by ART initiation, we compared neutralization score values in both cohorts only in individuals who had been on antiretroviral therapy for 6 months or more. With this analysis, we confirmed that neutralization potency and breadth after 6 months of ART were significantly higher in adults, *p* < 0.0001 (Figure 3b). On the other hand, the effect of long-term antiretroviral therapy on the induction of neutralizing responses after the first 6 months is unknown. To investigate this issue, we conducted a correlation analysis between neutralization score values and the duration of accumulated treatment, after the first 6 months, in both cohorts. No correlation was observed (Figure 3c).

On the other hand, the induction of broadly neutralizing responses has been generally associated with long periods of infection. However, in the present study, we did not observe any association between higher neutralization score values and longer times post-infection. In fact, neutralization score values in the adult cohort were higher despite having lower median post-infection times (8.8 years in adults and 10.8 years in children + adolescents (assuming all vertical infections occurred at birth or close to birth); Table 2). Taking into account that some individuals in the children + adolescent cohort could have been infected after birth through breast milk, the time post-infection in the 37 non-breastfed vertically infected individuals was also calculated. The mean time post-infection was still higher in these individuals than in adults (11.7 (1.4–14.7) years).

Another factor that could be responsible for the differences in neutralization breadth observed is the time elapsed from infection to treatment initiation. In this study, perinatally infected children and adolescents initiated ART at a median of 1.4 years post-infection. By contrast, the infected adults initiated ART at a median of 6.8 years post-infection (Table 2). Thus, viruses in the adult cohort had undergone more replication cycles prior to viremia suppression, which could have facilitated the induction of neutralizing responses of greater potency and breadth. In fact, neutralization score values were directly correlated with the time elapsed from infection to treatment initiation in both cohorts (adults (Spearman r = 0.2358, *p* = 0.0140) (Figure 3d) and children + adolescents (Spearman r = 0.3701, *p* < 0.0001) (Figure 3e)). Considering these observations, we aimed to study the effect of starting ART at times very close to infection (less than 3 months post-infection) on neutralization. In the cohort of children and adolescents, we observed that neutralization was almost undetectable if vertically infected children started ART within the first 3 months of life (Figure 3f). However, in the case of the adult cohort, this comparison could not be made because we did not have enough samples from individuals starting treatment in the first three months of infection.

### 3.3. Influence of CD4+ and CD8+ T-Cell Counts on Neutralization in Adults and Children with ART-Suppressed Viremia

**Figure 4:**
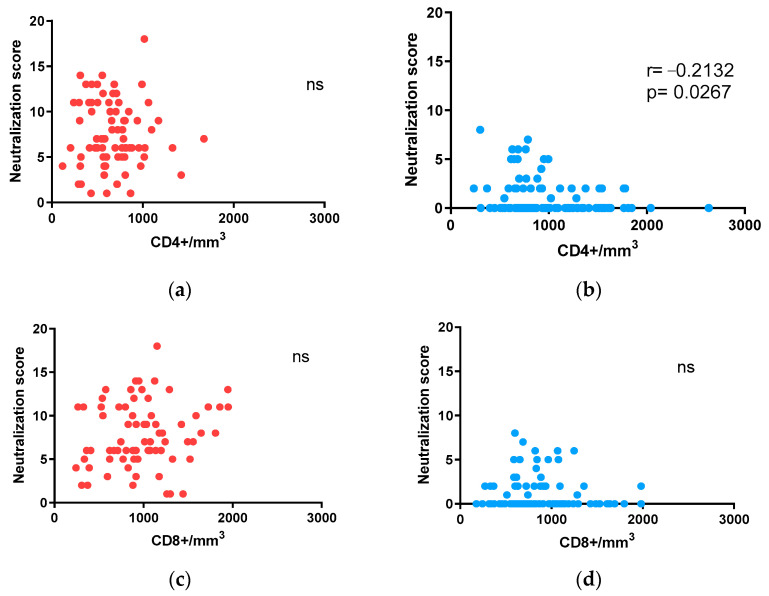
Neutralizing antibody response in relation to the number of CD4+ and CD8+ cells: (**a**,**b**) correlation of neutralization scores with CD4+ cells/mm^3^ in adults (**a**) and children + adolescents (**b**); (**c**,**d**) correlation of neutralization scores with CD8+ cells/mm^3^ in adults (**c**) and children + adolescents (**d**). Spearman r and *p* values (two-tailed) are indicated.

In some studies, a decline in overall CD4+ cell numbers was suggested to be linked with broad neutralization [12,27]. However, considering that viral load is a potent inducer of bnAbs and is inversely associated with CD4 levels [28], the inverse association between neutralization breadth and CD4+ cell counts has also been attributed to the corresponding viral loads. In the present study, we were able to analyze the impact of CD4+ cell count levels on neutralization without the effect of viral load owing to the fact that this study only includes individuals with undetectable viremia. In this scenario, a negative correlation between neutralization score values and CD4+ cell counts was observed in the children + adolescent cohort (Spearman r = −0.2132, *p* = 0.0267; Figure 4b). By contrast, no association was observed in the adult cohort (Figure 4a). Regarding CD8+ cell counts, no correlation was observed with neutralization score values in either of the cohorts (Figure 4c,d).

### 3.4. Impact of CD4+/CD8+ T-Lymphocyte Ratios on Neutralization in Adults and Children under 15 Years of Age with ART-Suppressed Viremia

CD4+/CD8+ ratios ≥1.0 have been associated with increased neutralization breadths within the first year of infection in the absence of ART, but this association was not observed in untreated chronically infected individuals. This observation suggests that, in the absence of ART, a less damaged immune system (CD4+/CD8+ ratio > 1) favors the induction of cross-neutralizing responses in recent infection but not in chronic infection [22].

**Figure 5:**
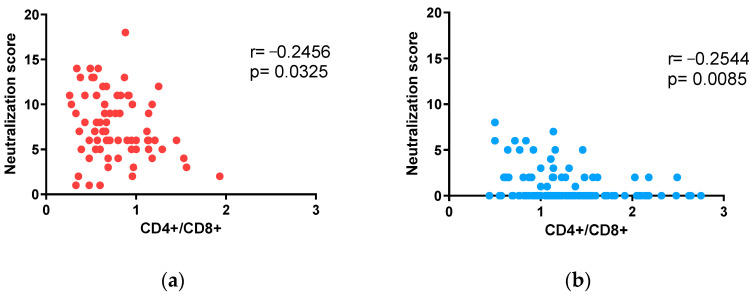
Influence of CD4+/CD8+ ratios in neutralization: (**a**,**b**) correlation of neutralization scores with CD4+/CD8+ ratios in adults (**a**) and children and adolescents (**b**). Spearman r and *p* values (two-tailed) are indicated.

However, the results obtained in the present study when comparing neutralization scores in ART-suppressed HIV-1-infected individuals contrast with what was previously described in untreated HIV-1-infected adults, both in recent and chronic infection [22]. In fact, neutralization score values in the children + adolescent cohort were significantly lower (Figure 3a) despite having higher CD4+/CD8+ than the adult cohort (1.20 and 0.68, respectively; Table 2). Notably, neutralization score values and CD4+/CD8+ ratios were inversely correlated in both cohorts (Spearman r = −0.2456, *p* = 0.0325 in adults and Spearman r = −0.2544, *p* = 0.0085 in children + adolescents; Figure 5).

### 3.5. Impact of nadir CD4+ T-Lymphocyte Counts on Neutralization in Adults and Children under 15 on ART

**Figure 6:**
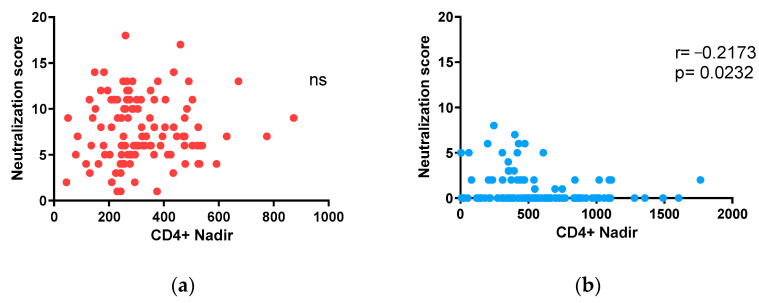
Neutralizing antibody response in relation to nadir CD4+ T-cell counts: (**a**,**b**) correlation of neutralization scores with nadir CD4+ T-cell counts in adults (**a**) and children and adolescents (**b**). Spearman r and *p* values (two-tailed) are indicated.

In the present study, the results show that in individuals on treatment with undetectable viremia, the induced neutralizing response depends on events that occurred at the beginning of the infection. Specifically, we found that the time elapsed from infection to the start of treatment was directly correlated with neutralization scores. Considering this observation, we decided to study the impact of CD4 nadir values on neutralization in both cohorts. In this analysis, we only observed a negative correlation between neutralization score values and CD4+ cell counts in the children + adolescent cohort (Spearman r = −0.2173, *p* = 0.0232; Figure 6b). However, no association was observed in the adult cohort (Figure 6a).

### 3.6. Neutralizing Antibody Response in Children under 10 Years of Age and Adolescents (10 to 15 Years of Age) with ART-Suppressed Viremia

**Figure 7:**
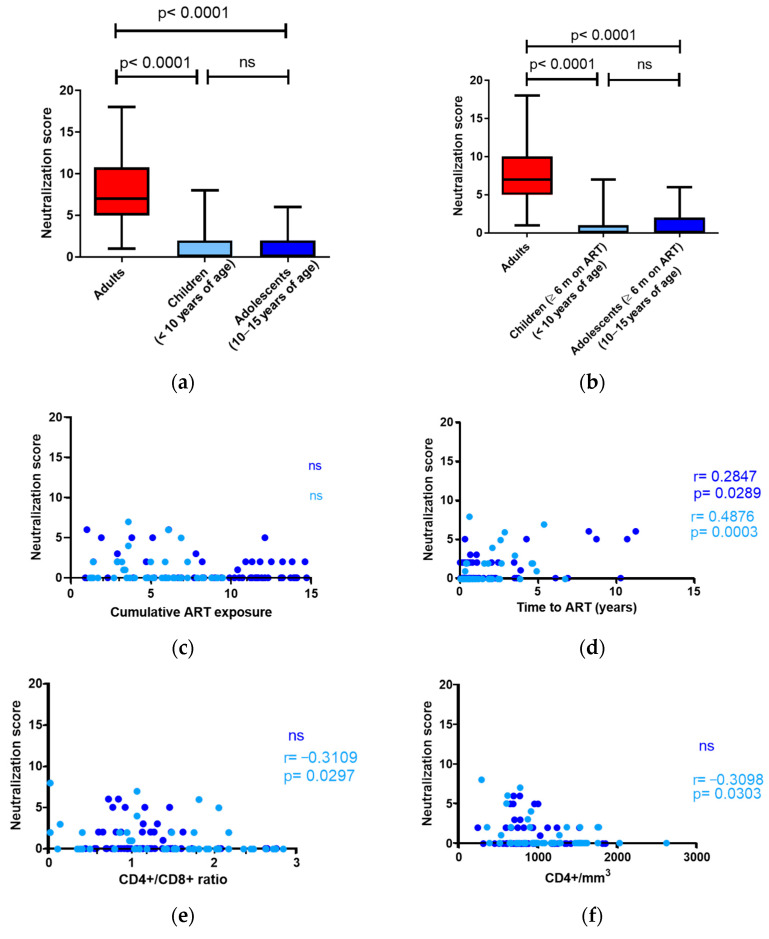

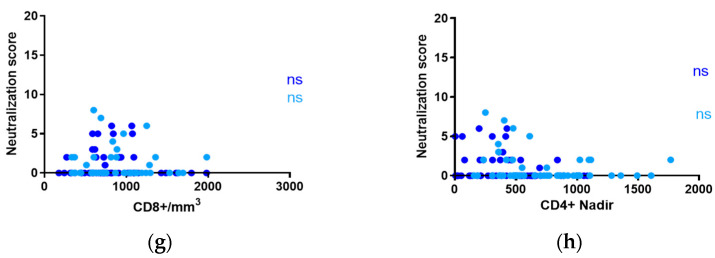
(**a**,**b**) Comparison of neutralization scores in adults, children, and adolescents. Horizontal bars within the box plots indicate the median for each group and standard errors of the means (SEMs). Significance between groups is indicated. Kruskal–Wallis test was used for comparison of the three groups; (**c**–**h**) neutralization antibody response in children and adolescents related to cumulative ART exposure, time to ART, CD4+/CD8+ ratios, CD4+T-cell counts, CD8+ T-cell counts, and nadir CD4+ T-cell counts. Spearman r and *p* values (two-tailed) values are indicated. Neutralization in children under 10 years of age is shown in light blue, and that of adolescents (10 to 15 years of age) is shown in dark blue. A *p*-value of <0.05 was considered significant.

Puberty, characterized by shifting sex hormone levels, the emergence of secondary sexual characteristics, and reproductive maturation, exerts significant influences on multiple organ systems, including the immune system. Considering this evidence, we decided to analyze the ability to induce cross-neutralizing responses against HIV-1 in ART-suppressed children under 10 and ART-suppressed adolescents between 10 and 15 years of age separately. Neutralization scores in both children and adolescents were significantly lower than those observed in adults, and no significant differences were observed between them (Figure 7a).

When analyzing the children and adolescent cohorts separately, we confirmed that neutralization scores were lower, even when individuals with less than 6 months of treatment were excluded from the analysis (Figure 7b). Neutralization scores did not correlate with the accumulated time on treatment (Figure 7c) or with CD8+ cell counts (Figure 7g), and there was a positive correlation with the time to treatment initiation (Figure 7d). The sample sizes were insufficient to replicate the analyses exclusively for non-breastfed individuals. The negative correlation between neutralization score values and CD4+/CD8+ ratios and CD4+ cell counts was only statistically significant in the children cohort (Figure 7e,f). CD4+ nadir values did not correlate with neutralization score values in children or adolescents (Figure 7h).

## 4. Discussion

bnAbs are crucial for an effective HIV-1 vaccine, yet despite extensive research, triggering their production through vaccination remains elusive. These potent antibodies are rarely generated naturally. Gaining insight into the factors that facilitate or hinder their development is imperative to inform the design of a vaccine capable of inducing bnAbs. This understanding is critical in advancing HIV-1 vaccine development.

In the present study, we confirmed that children and adolescents with treatment-suppressed viremia are capable of inducing broadly neutralizing responses. However, the magnitude of these responses is lower than that observed in adults. This observation contrasts with what has previously been observed in untreated infected individuals. In the absence of antiretroviral treatment, children exhibited a greater occurrence of broadly neutralizing responses than adults [15]. This difference has been attributed to differences in viral load kinetics at the early stages of infection. In adults, initial high virus levels rapidly drop 100–1000-fold, reaching a set point within weeks. In contrast, children show a slow virus decline, reaching a stable set-up around age 5, which results in prolonged exposure to high antigenic loads [15]. In the case of children who start treatment at birth, a typical starting viral load of 10^5^–10^6^ copies/mL drops to less than 50 copies/mL in 3–6 months [29], and therefore, antigenic stimulation at the early stages of infection is drastically reduced. As a result, 30% of vertically infected children who initiate ART within the first 3 months of age lose HIV-1-specific antibodies [30]. In our study, vertically infected children and adolescents who started treatment within the first three months of infection showed less neutralizing activity than children who started treatment later (Figure 3f). These findings related to neutralizing responses align with previously reported observations for HIV-1-specific antibodies [30]. In order to determine whether this absence of neutralizing antibodies is due exclusively to the early control of viremia or it also depends on specific characteristics of children’s immature immune systems, the same analysis needs to be performed in adults. Unfortunately, we were unable to conduct this analysis in the current study because we lacked a sufficient number of samples from adults who began treatment within the initial three months of infection.

As part of this study, we also analyzed neutralization data from children and adolescents separately and observed that, despite the hormonal changes associated with puberty, neutralizing responses were similar (Figure 7). We also again observed that the magnitude of the neutralizing responses did not depend on the time post-infection since both cohorts had similar neutralization score values despite the difference in median post-infection times between both groups (7.3 years in children and 12.6 years in adolescents; Table 2). Our results, together with those of previous reports [15,19], suggest that broadly neutralizing antibody (bnAb) induction depends on early infection dynamics. Following this hypothesis, in order to determine the impact of sex hormones on bnAb induction, neutralizing responses in individuals infected during childhood and adolescence should be investigated.

Despite the differences observed in the induction of broadly neutralizing responses between the two groups, we found similarities in all cohorts. In all cases, we found a positive association between neutralization score values and the time elapsed from infection to ART initiation and a negative correlation with CD4+/CD8+ ratios. On the other hand, we found no correlation with time post-infection, the accumulated time on treatment, or CD8+ levels.

Our findings confirm that children infected at birth and who have suppressed viremia as a result of antiretroviral therapy can indeed develop broadly neutralizing responses. However, these responses exhibit lower potency and breadth than those observed in adults. In addition, the induction of these responses is favored with more viral replication cycles prior to the start of treatment and, therefore, a greater antigenic stimulation before viremia suppression (longer times until ART initiation).

In conclusion, our research indicates that ensuring ample antigenic stimulation prior to initiating ART improves the capacity to produce long-lasting broadly neutralizing responses. This suggests that it is possible to induce these responses without the need for continuous vaccine exposure, as long as the initial antigenic stimulation is robust and sustained. These findings have significant implications for the development of effective vaccination strategies against HIV.

## Figures and Tables

**Table 1:** Frequencies of individuals with neutralization breadth.

		Children		Adults	
Neutralization Category	Neutralization Score ^a^	n	(%)	n	(%)
Elite	14–18	0	0	5	4.6
Broad	10–13	0	0	29	27.0
Cross	5–9	10	9.2	53	49.0
Weak or none	<5	99	90.8	21	19.4

^a^ A cumulative score was computed for each plasma, taking into account the neutralization scores against all six viruses using a previously described scoring system based on the percentage of inhibition of each virus of the panel using a single plasma IgG concentration (0.2 µg/mL) [25,26]. Thresholds for cumulative scores were determined to categorize plasma as elite, broad, cross, or weak/no neutralization.

**Table 2:** Clinical characteristics.

Characteristics	Children + Adolescents (<15 years)			Adults
	Children (<10 years)	Adolescents (10–15 years)	Total (<15 years)	
Total number of individuals	49	60	109	108
Number (%) of individuals by gender				
Male	23 (46.9)	24 (40)	47 (43.1)	94 (87.1)
Female	26 (53.1)	36 (60)	62 (56.9)	14 (12.9)
Number (%) of individuals by risk group				
Injecting drug user	0 (0.0)	0 (0.0)	0 (0.0)	4 (3.7)
Injecting drug user + homosexual	0 (0.0)	0 (0.0)	0 (0.0)	4 (3.7)
Bisexual	0 (0.0)	0 (0.0)	0 (0.0)	1 (0.9)
Heterosexual contact	0 (0.0)	0 (0.0)	0 (0.0)	24 (22.2)
Hemophiliac	0 (0.0)	0 (0.0)	0 (0.0)	1 (0.9)
Homosexual	0 (0.0)	0 (0.0)	0 (0.0)	69 (63.8)
Vertical transmission	49 (100)	60(100)	109 (100)	0 (0.0)
Unknown	0 (0)	0 (0)	0 (0)	5 (4.6)
Breastfeeding				
Yes	10	8	18	-
No	14	23	37	-
Unknown	25	29	54	108
Median (range) age (years) ^a^	7.3 (1.5–9.8)	12.6 (10–14.7)	10.8 (1.5–14.7)	42.0 (27.0–78.0)
Median (range) time post-infection (years) ^a^	7.3 (1.5–9.8) ^b^	12.6 (10–14.7) ^b^	10.8 (1.5–14.7) ^b^	8.8 (1.1–24.0)
Median (range) time to ART (years) ^a^	0.7 (0.0–6.7) ^b^	0.8 (0.0–11.3) ^b^	1.4 (0.0–12.6) ^b^	6.8 (0.1–23.9)
Median (range) time on ART (years) ^a^	5.0 (0.1–9.5)	10.45 (0.9–14.7)	6.9 (0.1–14.7)	1.3 (0.0–5.7)
Median (range) nadir CD4+ cells/mm^3 a^	696 (154–1765)	424 (5–1088)	488 (5–1765)	289 (45–873)
Median (range) CD4+ cells/mm^3 a^	1067 (300–2632)	814 (236–1846)	897 (236–2632)	640 (117–1673)
Median (range) CD8+ cells/mm^3 a^	780.5 (330–1982)	749.5 (176–1983)	754.5 (176–1983)	916 (243–1950)
Median (range) CD4+/CD8+ ratio	1.25 (0.50–2.50)	1.10 (0.40–2.80)	1.20 (0.40–2.80)	0.68 (0.26–1.93)
Viral RNA copies/mL plasma	<50	<50	<50	<50

^a^ At the time of sample collection. ^b^ Assuming infection occurred at birth or close to birth.

## Data Availability

Data are contained within the article.
